# Introgression Between Cultivars and Wild Populations of *Momordica charantia* L. (Cucurbitaceae) in Taiwan

**DOI:** 10.3390/ijms13056469

**Published:** 2012-05-24

**Authors:** Pei-Chun Liao, Chi-Chu Tsai, Chang-Hung Chou, Yu-Chung Chiang

**Affiliations:** 1 Department of Biological Science and Technology, National Pingtung University of Science and Technology, Pingtung 912, Taiwan; E-Mail: pcliao@mail.npust.edu.tw; 2 Kaohsiung District Agricultural Research and Extension Station, Council of Agriculture, Pingtung 908, Taiwan; E-Mail: tsaicc9017@yahoo.com.tw; 3 Research Center for Biodiversity, China Medical University, Taichung 404, Taiwan; E-Mail: choumasa@mail.cmu.edu.tw; 4 Department of Biological Sciences, National Sun Yat-sen University, Kaohsiung 804, Taiwan

**Keywords:** divergence time, effective population size, IM model, introgression, *Momordica charantia*

## Abstract

The landrace strains of *Momordica charantia* are widely cultivated vegetables throughout the tropics and subtropics, but not in Taiwan, a continental island in Southeast Asia, until a few hundred years ago. In contrast, the related wild populations with smaller fruit sizes are native to Taiwan. Because of the introduction of cultivars for agricultural purposes, these two accessions currently exhibit a sympatric or parapatric distribution in Taiwan. In this study, the cultivars and wild samples from Taiwan, India, and Korea were collected for testing of their hybridization and evolutionary patterns. The cpDNA marker showed a clear distinction between accessions of cultivars and wild populations of Taiwan and a long divergence time. In contrast, an analysis of eight selectively neutral nuclear microsatellite loci did not reveal a difference between the genetic structures of these two accessions. A relatively short divergence time and frequent but asymmetric gene flows were estimated based on the isolation-with-migration model. Historical and current introgression from cultivars to wild populations of Taiwan was also inferred using MIGRATE-n and BayesAss analyses. Our results showed that these two accessions shared abundant common ancestral polymorphisms, and the timing of the divergence and colonization of the Taiwanese wild populations is consistent with the geohistory of the Taiwan Strait land bridge of the Last Glacial Maximum (LGM). Long-term and recurrent introgression between accessions indicated the asymmetric capacity to receive foreign genes from other accessions. The modern introduction of cultivars of *M. charantia* during the colonization of Taiwan by the Han Chinese ethnic group enhanced the rate of gene replacement in the native populations and resulted in the loss of native genes.

## 1. Introduction

The tropical and subtropical bitter gourd, *Momordica charantia*, has been widely cultivated and domesticated for a long time [1]. This species, which is commonly used as a vegetable and an ingredient in traditional medicines, is cultivated in tropical and temperate China [2]. The phylogenetically related species *M. balsamina* (balsam apple) was used as a vegetable at least 500 years ago [3]. Ethnobotanical investigations reveal that cultivars were derived from the local wild types and that the encouragement of local variant populations, through plant utilization and habitat modifications, accelerated greater diversity among cultivated strains than among the local wild types [4]. The close association between cultivated strains and localities fixed the differences between strains and may have decreased the within-strain genetic diversity, which is the most significant difference between domesticated and wild species [5,6]. Individuals in the wild population found in Taiwan have small fruits morphologically different from the cultivars. Currently, the wild individuals are observed in markets in Taiwan but are primarily collected from wild populations rather than cultivated. Increasingly, more studies indicate that the wild bitter gourd contains antioxidants [7–9], which help to suppress the inflammatory responses [10–13] and lower blood-glucose levels in diabetes [14,15]. These studies of bitter gourd focus on the medical properties but rarely explore the origin, speciation, and hybridization/introgression of the local strains or varieties. Recent genetic evidence has shown that the Cucurbitaceae originated in Africa, and the genus *Momordica* was derived from South Africa, tropical Africa and tropical Asia [16,17].

Hybridization between crops and wild populations is common. Domesticated crops are usually artificially selected for adaptive traits, such as pathogen resistance [18] and higher fertility [19]. These domestication characteristics might be closely associated [20–22] with the role of “supergenes” [23]. Therefore, the hybrids of the domesticated crops and the wild populations would have greater fitness. If introgression occurs, the genes of the domesticated crop could quickly replace the genes of the wild populations by a small acceleration of immigration (e.g., human-mediated spread) [24] and result in genetic assimilation [23], which is the phenomenon of replacing a pure conspecific of one of the hybridizing taxa. Because the artificial hybridization between *M. charantia* cultivars and wild populations is successful when carried out by the Hualien District Agricultural Research and Extension Station (http://www.hdais.gov.tw/bred) for the purpose of improving the cultivars, natural hybridization is likely to have occurred in nature.

In addition, the introduction of the cultivars into Taiwan Island by the Han Chinese ethnic group began hundreds years ago at which time the wild population was already indigenous. Therefore, we wondered when the native wild population colonized Taiwan Island. Taiwan Island is a continental island located off the coast of Southeast Asia. It was lifted by orogenesis by tectonic compression of the Philippine Sea Plate and the Eurasian Plate *circa* (*ca.*) 6~5 million years ago (Mya) [25] or *ca.* 3 Mya [26]. The surrounding sea level change caused by the Pleistocene climate oscillation caused successive connection and disconnection of Taiwan Island to the Asian continent [27]. This process promoted the colonization and then isolation of several species from the Asian continent in Taiwan [28]. The native wild population is most likely the descendent of the ancient colonizers. In this study, we estimated the divergence time based on plastid DNA and nuclear markers. The divergence time estimated by maternally inherited plastid DNA could exclude the effect of pollen flow and reveal the time of colonization, while the divergence time estimated from the nuclear multilocus markers reflects the degree of isolation and the divergence between populations.

To evaluate the possibility of sympatric hybridization and to test the hypotheses of the relation of colonization of wild populations in Taiwan with the geological history, the chloroplast DNA (cpDNA) and the nuclear microsatellite loci are used as genetic markers to examine the genetic similarity of the cultivars and the wild populations and to trace their evolutionary histories by coalescent approaches. Because multiple strains of *M. charantia*, which might have distinct genetic compositions due to artificial selection, have been domesticated, different cultivated strains (instead of wild populations) are used to compare with the wild samples collected from Taiwan and other countries (India and Korea). In this study, we used evolutionary analyses to examine the hybridization and to explore the origin of the native population of *M. charantia*. This study demonstrates a crisis of genetic assimilation by introgression from a widespread crop.

## 2. Results

### 2.1. CpDNA Polymorphisms and Neighbour Joining Tree

**Figure 1:**
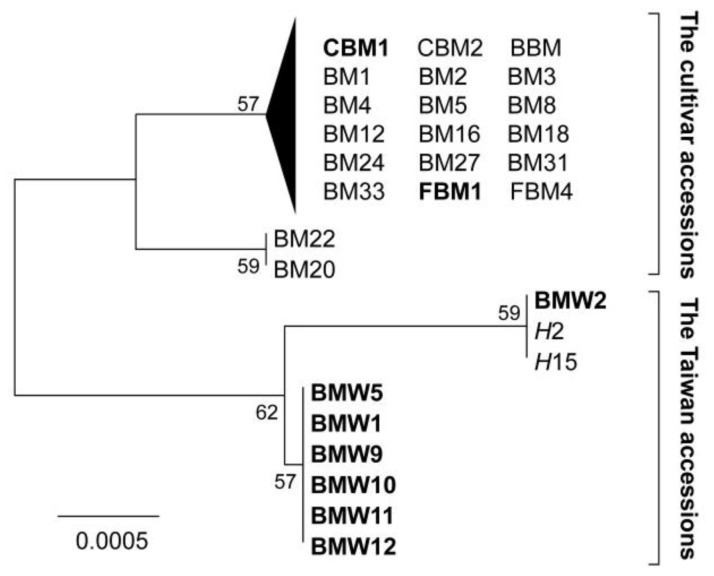
The neighbor-joining tree of the cultivars and wild samples of Taiwan, India, and Korea of *Momordica charantia,* inferred by the chloroplast *atp*B-*rbc*L spacer. Samples denoted in regular, bold, and italic are the cultivars, wild samples, and hybrids, respectively. The CBM1 and the FBM1 are wild samples from India and Korea, respectively. Each wild population and landrace cultivar variety included five to eight individuals with the same haplotype in the figure. Numbers between the branches are the supporting values for monophyly by 1000 bootstrap replications.

The cpDNA cp *atp*B-*rbc*L sequences from 164 individuals were aligned directly and five chlorotypes were found. The neighbor joining tree inferred from the cp *atp*B-*rbc*L sequences indicated that the wild population of Taiwan is distinct from other samples of *M. charantia*, including the cultivars and the wild samples of India and Korea, but grouped with the hybrid lines (Figure 1). The two major clades composed of samples of wild populations and hybrids from Taiwan and the samples of cultivars and wild samples of India and Korea are named “the Taiwan accession” and “the cultivar accession”, respectively. Because the cpDNA is maternally inherited, the interference of recent gene flow by pollen flow is eliminated. The BM22 (green cultivar) and BM20 (white cultivar) have identical genotype of cp *atp*B-*rbc*L, which means identical or similar maternal parents of these two cultivars. However, the parental lines of cultivars were not recognized and we cannot make sure whether these two cultivars are maternally identical by descents. The phylogenetic relationship displayed the long-term evolutionary differentiation between the Taiwan accession and the cultivar accession, which included wild samples from India and Korea. The grouping of hybrids indicated that the wild samples of Taiwan play the role of mother species (pollen receiver). The net average distance (*D*_A_) between the two accessions is 2.077 × 10^−3^ ± 1.414 × 10^−3^ (1000 bootstrap replicates under the maximum composite likelihood [MCL] substitution model). The divergence time (*T*) between these two accessions is 0.519 Mya (±0.354 Mya) using the formula *T* = *D*_A_/2*μ*, where *μ* is the mutation rate, while a longer time to the most recent common ancestor (TMRCA, 2.002 Mya, 95% HPD: 1.146 Mya~2.796 Mya) between the two accessions was estimated by using the BEAST. The large difference between the divergence time and the coalescent time between the accessions might be due to the high variance of the single-locus estimation or to the long time allowed for gene flow at the beginning of speciation [29].

In total, five haplotypes of 164 cp *atp*B-*rbc*L sequences were obtained from 29 lines of cultivars, hybrids, and wild populations of *M. charantia*. The aligned cpDNA *atp*B-*rbc*L data matrix was 954 bp in length. The sequences from this study can be found under the GenBank accession numbers: HE585487-HE585491. Four segregating sites were examined, and low genetic diversity was estimated. The nucleotide diversity (*π*) and genetic diversity (*θ*_W_) of total samples (including hybrids) is 0.00108 and 0.00100, respectively. The index of Tajima’s *D* of the total sample is 0.1977 and is not significant so the null hypothesis of neutral evolution cannot be rejected. Genetic diversity indices of two accessions are listed in Table 1.

### 2.2. Nuclear Microsatellite Polymorphisms

**Figure 2:**
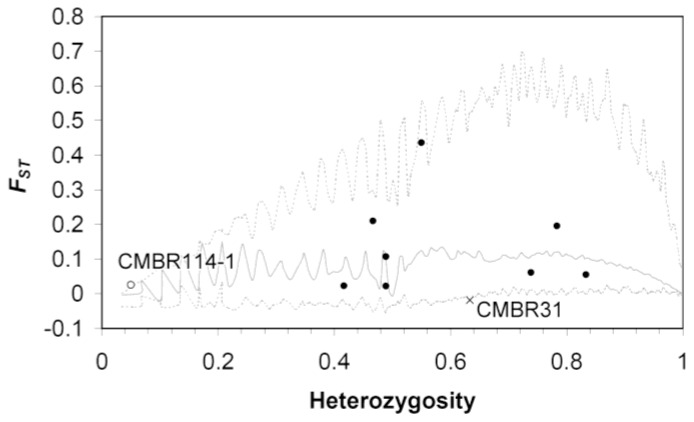
Distribution of population genetic differentiation index *F*_ST_ as a function of heterozygosity by simulation, while considering the neutral markers only, and forcing the mean *F*_ST_ under a stepwise mutation model. The dashed lines and the solid line represent the 0.95 quantiles and the median, respectively. Each dot represents a microsatellite locus; loci above the 0.95 quantile line (upper dotted line) were classified as under divergent selection, while those below the lower dotted line were classified as loci under balancing selection.

Eleven of 72 primer sets developed from Ritschel *et al.* [30] were successfully amplified in all samples of this study and 12 loci were obtained from these 11 loci, which were all confirmed by direct sequencing. Except for the two loci (CMBR114-1 and CMBR114-2) that were amplified from the primer set CMBR114, one locus was obtained from each of the primer sets. However, two loci (CMBR114-2 and CMBR145) are monomorphic. The neutrality of evolution of each locus was tested by detecting the *F*_ST_-outliers by criteria of 99% confidence intervals (CI). CMBR114-1 and CMBR31, which are thought to have evolved under positive selection (extremely high *F*_ST_) and balancing selection (extremely low *F*_ST_), respectively (Figure 2), were detected as the *F*_ST_-outlier loci. The locus CMBR114-1 is monomorphic in wild population of Taiwan samples but polymorphic in cultivars. The fixation of CMBR114-1 in Taiwan accession is probably reasoning for the extremely high *F*_ST_ among accessions. We estimated the genetic diversity by expected heterozygosity (*H*_exp_) and genetic diversity index (*θ*) of the remaining eight microsatellite loci for each accession and obtained a wide range of diversity for each locus: for the cultivar accession *H*_exp_ ranges from 0.097 to 0.738, and *θ* ranges from 1.506 to 5.186; and for the Taiwan accession *H*_exp_ ranges from 0.264 to 0.791, and *θ* ranges from 1.506 to 2.527 (Table 2). Note that the estimates for the cultivar accession are based on the genetic diversity of cultivated strains, but the estimates for the Taiwan accession represent the genetic diversity of the wild population.

### 2.3. Genetic Mixing Between the Cultivars and the Wild Population of Taiwan

**Figure 3:**
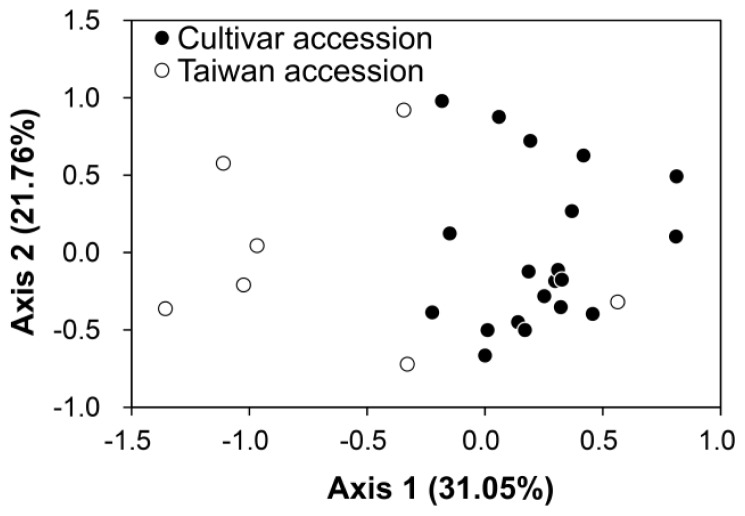
Population structure estimated by the principle coordinate analysis (PCoA). Scatter plot of the first axis (31.05% explanation) and the second axis (21.76% explanation) of the PCoA based on variations of 8 microsatellite loci (excluding the *F*_ST_ outlier loci CMBR31 and CMBR114-1). The black and white dots indicated the individuals from “the cultivar accession” and “the Taiwan accession”, respectively.

**Figure 4:**
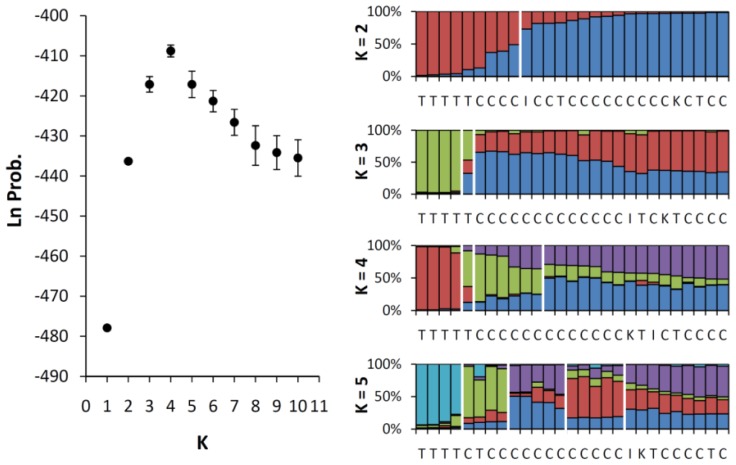
Genetic subdivision inferred using the Bayesian-clustering method implemented in the STRUCTURE program [31] for eight neutral microsatellite loci. The left plot is the log of the estimated posterior probability of *K*, which indicates a best fit of *K* = 4; the right panels are individual genotypes grouped by *K* = 2~5; the marks C, T, I, and K of each individual plot indicate the cultivar samples, the wild samples of Taiwan, India, and Korea, respectively.

The AMOVA results showed that most of the genetic variation results from within-accession variation (99.44%), which indicates that the genetic differentiation between the two accessions is not significant (fixation index *R*_ST_ = 0.0056, *P* = 0.6149). From the review of genetic differentiation by Storz *et al.* (2005), genetic differentiation will be significant if the genetic variation of one of two populations (here, the accessions of *M. charantia*) is significantly lower than the other. In our study, the high percentage of genetic variation within accessions combined with insignificant genetic differentiation between accessions seems to imply that the degree of genetic variation of the Taiwan accession, the wild bitter gourd, is not very different from the genetic variation among multiple cultivar lines of *M. charantia*. However, the accession-specific *R*_ST_ values of the two accessions are 0.00139 and 0.01758 in the cultivar accession and the Taiwan accession, respectively. The difference in *R*_ST_ between the two accessions is larger than 10-fold, indicating a larger genetic differentiation of the wild population, which seems unreasonable because different sources (lines) of the cultivars have been suggested to have higher differentiation than the local populations. Therefore, clustering patterns of these samples were rechecked with PCoA. We also used the PCoA to reassign the clustering of collected samples without any assumption of prior classification. The result of the PCoA revealed mixture patterns of individuals between the two accessions in two axes with 52.81% explanation (31.05% and 21.76% for the first and the second axis, respectively) (Figure 3). This result implies that there is small genetic differentiation with certain admixture between the two accessions, which is consistent with the result of the AMOVA (Table 3). To understand whether there are cryptic groupings in certain cultivated lines of *M. charantia* and wild populations in Taiwan, we performed another assignment test based on the genetic composition with the assistance of STRUCTURE v. 2.2 [31]. In the STRUCTURE analyses, both admixture and no admixture models were tested in 10 replicates for *K* = 1~10, where *K* is the grouping number, and the likelihood values of each *K* were evaluated. The best clustering was K = 4 (the highest likelihood, lnL = −362.7, estimated lnProb. = −411.6, Figure 4), but the grouping was not consistent with the grouping of the cpDNA tree. This result indicates that the genetic composition of nuclear DNA is not assigned as well as the grouping of maternally inherited cpDNA and implies contributions of pollen flow to genetic admixture.

### 2.4. Evidence of Asymmetric Introgression Inferred by the Isolation-with-Migration Model

**Figure 5:**
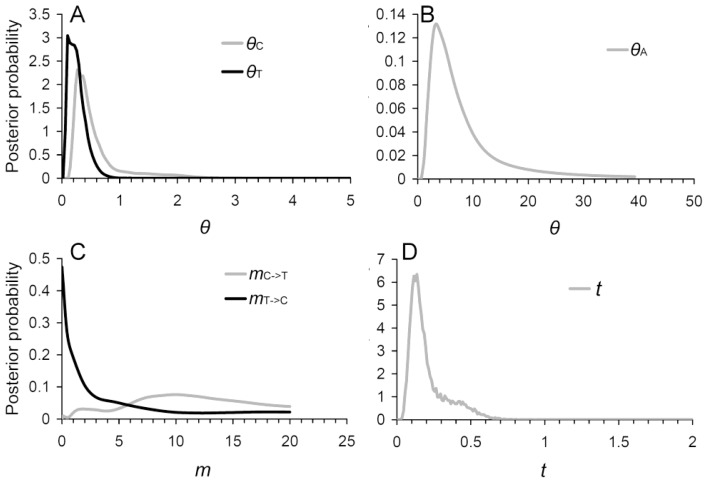
Marginal posterior probability density distribution of (**A**) the current effective population size of the cultivar accession and the Taiwan accession; (**B**) the ancestral population size; (**C**) migration rates of the cultivar accession and the Taiwan accession; and (**D**) divergence time of these two accessions, each scaled by the geometric mean of the mutation rates of all loci analyzed.

The isolation-with-migration (IM) model [32] that allows for the population size change and gene flow after divergence was used for examining the degrees and directions of gene flow between the cultivar accession and the Taiwan accession as well as their divergence time. For fitting the assumption of the IM model, two putatively positively selected genes were removed in this analysis. According to the eight neutral loci, the unscaled divergence time *t* = 0.135 (95% CI: 0.065–0.545) has the highest posterior probability (Figure 5D) estimated by the program IMa [33]. If we used a rough mutation rate of 7.47 × 10^−6^ per year for microsatellites, the divergence time is *ca.* 18.07 kilo-years ago (kya) (95% CI: 8.70 kya~72.96 kya), which is much smaller than the 0.519 Mya estimated from cpDNA *atp*B-*rbc*L spacer. The difference in divergence time inferences might be due to the intrinsic factor of rapid evolutionary rate of microsatellites compared with cpDNA, resulting in faster coalescence of the wide ranges of evolutionary rate of microsatellites, and extrinsic factors such as the pollen flow. In addition, we also noticed that the posterior probability is zero when *t* = 0, indicating that these two accessions are truly diverged. This result does not support the inference of the admixture pattern of genetic structure analyses (PCoA, AMOVA, and STRUCTURE).

In the analysis of gene flow, the direction from the cultivar accession to the Taiwan accession has a maximum posterior probability of migration rate *m* = 9.95 while the opposite direction of migration has a maximum posterior probability of *m* = 0.01 (Table 4 and Figure 5C). However, this result cannot be used to reject the null model *m*_T→C_ = 0 (Log-likelihood ratio 2LLR = 0.002, *P* = 0.967), indicating a unidirectional gene flow (or introgression) from the cultivar accession to the Taiwan accession (Table 4). The estimated effective population size of the ancestor (*θ*_A_ = 3.3572) is considerably larger than both current populations of the cultivar accession (*θ*_C_ = 0.3314) and the Taiwan accession (*θ*_T_ = 0.1767) (Figure 5A,B). The fourfold larger population size of the cultivar accession compared with the native wild population of Taiwan is most likely due to the multiple sources of cultivated lines. However, the equal effective population sizes of the cultivar and the Taiwan accessions (*θ*_C_ = *θ*_T_) cannot be rejected (2LLR = 0.0056, *P* = 0.470), which suggests that these two accessions preserved equal weighted genetic diversity (Table 4). Both the estimation of the equal population size and unidirectional gene flow implied that the wild population of Taiwan receives a large amount of polymorphism from the cultivar accession, and the abundant foreign polymorphism received by the Taiwan population explains its high accession-specific *R*_ST_. Except for the model *m*_T→C_ = 0 and *θ*_C_ = *θ*_T_, the other nested models were rejected at the level of *P* < 0.05 by the log-likelihood ratio tests (Table 4).

### 2.5. Historical and Contemporary Gene Flow

**Figure 6:**
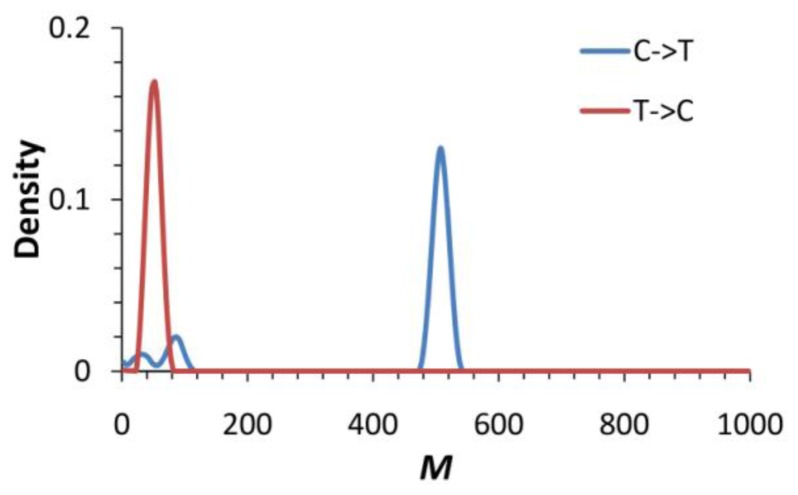
Posterior distribution of migration rate (*M*) over all loci between the cultivar accession and the Taiwan accession estimated by using the MIGRATE-n [34] analysis.

Patterns of historical gene flow estimated by MIGRATE-n v. 3.0 [34] are consistent with the inference by IMa [33] that the degrees of historical gene flow from the cultivar accession to the Taiwan accession (*M*_C→T_ = 507.5, 97.5% CI: 475.0~535.0, where the suffixes C and T indicate the cultivar accession and the Taiwan accession, respectively) are larger than the opposite direction (*M*_T→C_ = 52.5, 97.5% CI: 25.0~75.0) (Table 5 and Figure 6). The migration rate *M* estimated by using the MIGRATE-n [34] was scaled by *μ* and such high and significant different values of *M* indicated the historical asymmetric gene flow (*i.e.*, introgression) between two accessions. This result is consistent with the inference of unidirectional gene flow by IMa analysis. Such asymmetric gene flow was also detected in the recent migration events by the assignment test of BayesAss [35] that (1) the inter-accession gene flow was detected at a frequency (rate) of migrations of 0.166 (95% CI: 0.00787~0.325) between accessions under 10^7^ simulations, and (2) the rate of gene flow from the cultivar accession to the Taiwan accession is 0.0707 (95% CI: 0.0026~0.1963), and the opposite direction is 0.0151 (95% CI: 0.0005~0.0522) (Table 5). The rate of migration within accessions (intra-accessions gene flow) was 0.9849 (95% CI: 0.9478~0.9995) and 0.9293 (95% CI: 0.8037~0.9974) in the cultivar accession and the Taiwan accession, respectively. The smaller degree of gene flow between accessions than the intra-accession gene flow also implied more or less isolation (barrier) of reproduction between accessions in spite of the secondary contacts due to agricultural reasons.

## 3. Discussion

The first aim of this study was to test whether the cultivars of bitter gourds are genetically differentiated from the wild populations of Taiwan or whether these two accessions are a genetic mixture. Although the cultivars and the wild populations of Taiwan are taxonomically defined as the same taxon, which is also supported by AMOVA (Table 3), PCoA (Figure 3), and Bayesian clustering analysis (STRUCTURE) (Figure 4) by nuclear SSR, the phylogenetically distinguished accession of the wild population in Taiwan from cultivars indicated that the wild population of Taiwan has been differentiated from the inland populations (including two lines of wild populations from India and Korea) for a long time. The inland populations of Asia are thought to be sources of the domesticated lines of bitter gourds. Because of the maternally inherited characters, the cpDNA tree reflects the faithful phylogenetic relationships without the interference of recent pollen flow. The cpDNA phylogeny revealed different evolutionarily significant units between the cultivar accession and the wild population of Taiwan (the Taiwan accession), and the incongruent grouping between the cpDNA and nuclear SSR implied the recent introgression between the two accessions due to pollen flow. The domestication history of *M. charantia* spans thousands of years [1], and the morphological transitions in the fruit size, shape, color, taste, and many other traits are not reflected by the selected loci used in this study. Although only a small number of the SSR loci were used, which cannot completely represent the whole genome variation, the undifferentiated patterns detected in eight neutral loci implied that these morphological transitions are not the outcome of species (population) divergence but a consequence of selection (either natural or artificial) for specific traits. Accordingly, the impact of the translocation of diversified cultivars on wild populations might be encrypted in phenotypes and therefore worth further consideration.

There are two explanations for the admixture pattern of the genetic composition of the two accessions: (1) incomplete divergence by sharing abundant common ancestral polymorphisms and (2) gene flow after divergence. Although the posterior probability of the splitting time (*t*) estimated by using the IMa analysis is zero at minimum *t* (Figure 5D), the high estimates of historical *M* by MIGRATE-n analysis suggest that the divergence between the two accessions is not complete. In other words, the divergence of nuclear genomes was begun at *ca*. 18 kya, but the speciation process is still progressing. This age roughly matches the time of LGM, during which the climate was colder and drier than at present, and the distribution of the ancestors of these two accessions might be restricted to refugia. At that time, the shallow shelf of the Taiwan Strait was exposed and formed a land bridge between mainland Asia and Taiwan Island. The LGM at approximately 18 kya has been suggested to be a major factor in influencing species evolution (e.g., speciation) in Taiwan. After the LGM (*i.e.*, postglaciation), the geographic barrier of the Taiwan Strait isolated the island populations from mainland China. The perfect match of the divergence time of the two accessions and the age of the LGM suggests a relation between the evolutionary history of *M. charantia* and the geohistory of Southeast Asia. In addition, the shorter divergence time estimated by microsatellites than cpDNA implied the continuous gene transport by pollen flow between accessions after the divergence of maternally inherited materials since the middle Pleistocene, *i.e.*, a long-term process of speciation (divergence).

Furthermore, the large ancestral population size (*θ*_A_ = 3.357) of the two accessions (as inferred by the coalescent approach under the IM model) is in contrast to the relatively small population sizes of the derived accessions, reflecting a prominent stochastic effect of lineage sorting among multiple loci [36]. The large ancestral population size also implied that these two accessions share an abundant genetic background with their ancestors before divergence. Considering the recent divergence time (*ca*. 18 kya) inferred by microsatellites and that the cultivar accession is not native to Taiwan Island, the native population of the Taiwan accession most likely colonized Taiwan during the late Pleistocene glaciations (*i.e.*, the LGM). Certain examples, such as lizards [37], earthworms [38], butterflies [39], and crabs [40], indicated that the LGM provided a chance for colonization from mainland Asia to Taiwan, and the subsequent postglacial sea level rise caused the isolation of colonizers from the continental populations and resulted in genetic differentiation. However, the gene flow seems to have continued after the separation of Taiwan Island and mainland Asia as inferred by MIGRATE-n [34] and BayesAss [35]. The past and current gene flow between accessions in the coalescent process illustrates a gradual process of speciation of the local population of Taiwan instead of a sudden allopatric isolation [41]. This inference is also supported by the wide range of the 95% CI of the divergence time (8.70 kya~72.96 kya) inferred by IMa. The postglacial climate warming provided better growth conditions for both continental and island populations. Therefore, geographic isolation and local adaptation might be two key factors contributing to the divergence (speciation) of the two accessions.

In addition, the relatively smaller effective population size of the wild population of Taiwan (approximately a quarter of the cultivars) is truly a reflection of the locally restricted distribution in Taiwan. Reduced genetic diversity is a common feature of domesticated genomes because of a “genetic bottleneck” caused by the domestication process [42] or a selective sweep for local genomic regions surrounding the locus of human-mediated selection [43]. However, the cultivars revealed higher genetic diversity and lower genetic differentiation (*R*_ST_) than the wild population of Taiwan, which implied multiple sources of different cultivated strains (*i.e.*, independent domestication events from multiple local ecotypes instead of progressing domestication from one single strain) followed by artificial hybridization for breeding purposes. Multiple independent domestication events from local strains might have resulted in the rapid fixation of domestication-related genes while most unlinked loci of the genome retained the degree of diversity that was present before the initial domestication. This mechanism might account for the high genetic diversity of the nuclear microsatellites of the cultivars of *M. charantia*. Another possibility is that crops restored diversity through recent gene flow from wild populations after domestication [44]. We are not sure whether the large genetic diversity was contributed by the introgression from wild populations to cultivars, either at the initial stages of domestication or recurrently because of the lack of wild inland samples of *M. charantia*. However, the assignment tests by Bayesian clustering analysis (Figure 4), which infers the recent gene flow, revealed genetic admixture between the wild population of Taiwan and cultivars. The gene flow from the wild population of Taiwan to the cultivars is inferred to be rare (*m*_T→C_ = 0.01) by the IM model, and the hypothesis of no migration (*m*_T→C_ = 0) cannot be rejected (Table 4). On the other hand, the opposite direction of gene flow is higher (*m*_C→T_ = 9.95), suggesting that the genetic admixture is a result of strong introgression from cultivars to wild populations of Taiwan. This inference is also supported by the analyses of historical and current gene flow by using the MIGRATE-n [34] and BayesAss [35], respectively, which show a continuing asymmetric introgression from cultivars to the wild population of Taiwan. The high migration rates indicate the lack of reproductive isolation between two accessions and different capacities to receive foreign genes. The secondary contact by human mediated introduction (e.g., cultivation) would intensify such asymmetric gene flow, which could be occurring because of the increase in the recent migration rates (Figure S1), although estimates of the times of gene flow are sometimes inaccurate [45,46].

## 4. Experimental Methods

### 4.1. Sample Preparation

Seeds or adult leaves of both *M. charantia* cultivars and wild populations were collected from the field by our investigators or by exchange with cooperating laboratories. Most of the cultivars were provided by the National Plant Genetic Resources Center of the Taiwan Agricultural Research Institute, Council of Agriculture of Taiwan. The lines of varieties are listed in Table 6. Five to eight individuals of each wild population from Taiwan, India, and Korea and the white and green landraces of *M. charantia* were grow or collected freshly matured leaves, purified genomic DNA, sequenced the *atp*B-*rbc*L intergenic spacer of cpDNA, and genotyped SSR, independently, to test the homogenization. In total, 164 individuals were tested on this study and listed in Table 6. Soaked seeds of *Momordica* lines were incubated for four days in 30 °C to allow them germinate. Subsequently plants were cultivated in thermostatic chamber in 25 °C for two weeks and then transplanted into greenhouse conditions for the follow-up study. Freshly matured leaves were collected for molecular experiments.

### 4.2. Molecular Techniques

Total DNA was extracted from leaves according to the protocol of the Plant Genomic DNA Extraction Miniprep System (Viogene, Taipei, Taiwan). Following the commercial protocol, 100 mg fresh leaves from each individual were used for purifying genomic DNA. Totally, extracted genomic DNA from 164 individuals was dissolved in 200 μL of TE buffer (pH 8.0) and stored at −20 °C for further experiments. Seventy-two primer sets developed from *Cucumis melo* L. [30] were tested for our samples by PCR, isolation, and sequencing and 12 microsatellite loci with perfect tandem repeat sequences were found. Eleven microsatellite primer sets, which totally extract 12 microsatellite loci, were successfully amplified in all cultivated and wild samples of *M. charantia* (Table 2). The thermocycling profiles were as follows: initial denaturing at 94 °C for 5 min, followed by 30 cycles of 40 s for denaturation at 94 °C, 1 min annealing at the optimized annealing temperature (Table 2), 1 min extension at 72 °C, and a subsequent final extension for 10 min at 72 °C. We chose certain amplicons for direct sequencing to confirm the polymorphic patterns of tandem repeats. The other amplicons were scored using an ABI 3730 for genotyping, and GeneMapper 3.7 software (Applied Biosystems, USA) was used for fragment analysis. The PCR amplification of sequences of the cpDNA *atpB-rbcL* intergenic spacer was performed using the Gene Amp^®^ PCR System 9700 (Applied Biosystems, CA, USA) and the primers atpB-107 and rbcL-188 were derived from Chiang *et al.* [47]. The PCR mixture (50 μL) contained 500 mM KCl, 15 mM MgCl_2_, 0.1% Triton X-100, 100 mM Tris-HCl (pH 8.3), 2 mM dNTPs, 2 μM primers (forward and reverse), 20 ng template DNA, 1 μg RNase, and 0.5 U Taq polymerase (Clontech Laboratories, Inc., CA, USA). The program for amplifying *atpB-rbcL* was as follows: initial denaturation at 94 °C for 5 min followed by 35 cycles of 40 sec at 94 °C, 1 min annealing at 55 °C, 1 min at 72 °C and a subsequent 10 min final extension at 72 °C. The purified PCR products were sequenced in both directions using a BigDye^®^ Terminator v 3.1 Cycle Sequencing Kit (Applied Biosystems, CA, USA), the ABI 377XL automated sequencer (Applied Biosystems, CA, USA) and the ABI PRISM^®^ 3700 DNA Sequencer (Applied Biosystems, CA, USA) at the Genomics BioSci & Tech. Co., Ltd., Taipei, Taiwan.

### 4.3. Data Analyses

#### 4.3.1. Genetic Diversity and Neutrality Tests

The amplified cpDNA sequences from 164 individuals were aligned directly by ClustalX [48] and edited for further analyses using BioEdit ver. 5.0.6 [49]. Chlorotype sequences were generated using DnaSP ver. 4.0 [50]. The average number of mutations between pairs (*π*) and the index of genetic diversity estimated by segregating sites (*θ*_W_) were calculated using DnaSP ver. 4.0 [50]. The Tajima’s *D* neutrality tests [51], estimated by calculating *π* and *θ*_W_, were performed using DnaSP ver. 4.0 [50].

#### 4.3.2. Phylogenetic Analyses of Chloroplast DNA

Neighbor-joining phylogeny and Bayesian phylogenetic relationships of chlorotypes were constructed using MEGA v. 5.05 [52]. The settings for the MCL model [53,54] for substitutions and pairwise deletion for indels, and the amount of support for monophyly evaluated by 1000 replicates in bootstrap resampling were used for the NJ tree reconstruction. For estimating the coalescent time of the cultivars and the wild samples of *M. charantia*, the phylogenetic Bayesian Markov chain Monte Carlo simulations were performed using BEAST v. 1.6.1 [55]. The Markov chains were run for 10 million generations and were sampled every 1000 generations, with the first 1000 samples discarded as burn-in. The HKY model was used based on the evaluation by using the corrected Akaike information criterion (AICc) and Bayesian information criterion (BIC) with the assistance of MEGA v. 5.05 [52]. The Yule’s birth-rate model with gamma distribution was set for prior trees with a randomly generated starting tree. Three replicates were run and combined for estimating the divergence time and the TMRCA with a setting of the substitution rate as 2 × 10^−9^ per site per year [56].

#### 4.3.3. Neutrality Test by Detecting Outlier Loci for Microsatellite DNA

Before extending our analysis to genetic structure and speciation model tests, it was necessary to perform a neutrality test on the polymorphic microsatellite loci (*i.e.*, the loci with any repeat-number variation) to ensure the exclusion of the influence of natural selection. We used the method of Beaumont and Nichols [57] to identify the *F*_ST_ outlier for each locus individually. The idea of outlier loci is based on the hypothesis of extremely high *F*_ST_ between populations for positively selected loci (the positive outliers) than for the neutral loci and a reduced *F*_ST_ between populations for loci under balancing selection (the negative outliers). The *F*_ST_ outlier approach is performed by LOSITAN [58] using two strategies: (1) we ran LOSITAN once to calculate the mean *F*_ST_ by empirical data followed by forcing the simulation according to the mean *F*_ST_; (2) we ran LOSITAN as in strategy 1 but simulated by removing the loci outside the 99% CI as recommended by Antao *et al.* [58]. The stepwise mutation model (SMM) was used for 100,000 simulations. Each strategy was run three times to obtain a converged inference for ensuring the accuracy of estimation.

#### 4.3.4. Genetic Diversity and Genetic Structure

The expected heterozygosity and index of genetic diversity (*θ*) of the “neutral” microsatellite loci were estimated using the stepwise mutation model (SMM) by Arlequin v. 3.0.1 [59]. The genetic structure between the two accessions was evaluated by an analysis of molecular variance (AMOVA) [60], a PCoA, and the assignment test by Markov chain Monte Carlo (MCMC) simulation performed by using the STRUCTURE program [31]. The candidate adaptive loci were excluded in these analyses. The AMOVA was performed with Arlequin v. 3.0.1 [59] based on SMM [61]. Based on the phylogenetic relationships reconstructed by cpDNA, two accessions (the cultivar accession and the Taiwan accession) were set. Between-accession and within-accession variations were estimated as a percentage of the total genetic variation. Statistical significance for evaluating fixation indices (*R*_ST_) from random variation (no genetic differentiation) was tested using 1000 permutations. PCoA was performed using GenAlEx 6.3 [62] in a covariance standardized setting. In the PCoA, polymorphic loci were treated as independent traits to estimate the eigenvalues of the switched axes. The three-dimensional PCoA plot was redrawn with the assistance of JMP v. 7.0 [63]. The genetic structure was further examined by using the Bayesian clustering method implemented in STRUCTURE v. 2.2 [31]. Both admixture and no admixture models were used. The posterior probability of the grouping number (*K* = 1~10) was estimated by using the MCMC method with 10 independent runs to evaluate the consistency of the results using 3 million steps with a 500,000-step burn-in for each run. Better grouping numbers were evaluated by log estimates of the posterior probability of *K*.

#### 4.3.5. Isolation with Migration (IM) Model Test

After the candidate loci under selection were excluded, the remaining eight neutral loci, which were detected in the mode of SMM, were used for the IM analysis [32]. We performed the IM analysis by using the IMa program [33], in which the nested models were tested by using the likelihood-ratio test (LRT) to test whether the speciation mode of divergence with gene flow is fitted to the cultivar accession and the Taiwan accession of *M. charantia*. Four specific questions were addressed to resolve questions concerning the split time, migrations (gene flow), and population sizes: (1) Are the cultivar accession and the Taiwan accession really divergent? (2) Is there a change in population size after their divergence? (3) Has any migration occurred since the divergence of two accessions? (4) How do the degrees of migration (gene flow) differ between the two accessions? The IMa uses the MCMC sampling strategy to simulate parameters (scaled by mutation rate) of population size (*θ*_A_ = 4*N*_A_*μ; θ*_1_ = 4*N*_1_*μ; θ*_2_ = 4*N*_2_*μ*, where *N* is the effective population size, and *μ* is the mutation rate), migration rate (*M*_1_ = *m*_1_/*μ*; M_2_ = *m*_2_/*μ*, where *m* is the unscaled migration rate), and divergence time (*t* = T/*μ*, where *T* is the unscaled divergence time). The model of molecular evolution employed was the SMM with an inheritance scalar of 1 for the nuclear microsatellite loci. MCMC runs were carried out with 100,000-step burn-in followed by 10 million iterations of the Markov chain. Because the mutation rate for the microsatellite loci might vary and cannot be determined, the default mutation rate (*μ* = 1) was set. Most effective sample sizes (ESS) for the microsatellite loci are never higher than 50; thus, the lack of trends in the parameter plot for *t* was used to determine whether convergence had been reached. Log-likelihood ratio (2LLR) tests were performed to test the significance of various nested models (null models) implemented in the IMa. We used null models that included various combinations of equal population sizes of the ancestral (*θ*_A_) and the current populations (*θ*_C_ for the cultivar accession and *θ*_T_ for the Taiwan accession), equal bidirectional migrations (*m*_C→T_ = *m*_T→C_), and a fixed value of zero for migrations (Table 4). The 2LLR values approximately fit a *χ*^2^ distribution, and the associated probabilities were calculated with a *χ*^2^ distribution, as recommended [33].

#### 4.3.6. Inferring the Past and Recent Introgression

The historical and recent gene flows were estimated with the assistance of the MIGRATE-n v. 3.0 [34] and the BayesAss programs [35], respectively. The MIGRATE-n was executed by sampling genealogies under assumptions of constant population size and migration rate over coalescent time to obtain the maximum likelihood of migration rate. The Brownian mutation model and the prior distribution of variable *θ* with variable migration rate were incorporated into the Bayesian approach in the MIGRATE algorithm. Varying mutation rates among loci were adopted by empirical estimation (rates 0.4~1.8 per locus). The *F*_ST_ estimates were used as starting values for the initial analysis. For all other analyses, the ending parameters of the previous run were used as the starting values for the next run until results equilibrated at approximately the same values. We used the settings of three long chains with 15 million sampled, 15,000 recorded, and 25% discarded at the beginning of the chain. Adaptive heating was applied with temperature specifications of 1.00, 3.71, 9.14 and 20.00 to assure convergence, and the last three replicates were combined for estimation.

The pattern of recent gene flow between accessions was inferred by using the Bayesian inference with the assistance of BayesAss v. 1.3 [35]. This approach allows a dataset free from Hardy-Weinberg or migration-drift equilibrium. Three independent MCMC runs for 10^7^ iterations with every 2000 iteration of sampling were performed, and the first 10% iterations were discarded as burn-in. Three delta parameters of allele frequency, migration, and inbreeding were set as 0.15, 0.15, and 0.20, respectively, based on the preliminary runs.

## 5. Conclusions

The unresolved genetic structure and the high migration rates between cultivars and the wild populations of Taiwan of *M. charantia* suggest incomplete divergence and a high capacity for gene exchange. In fact, artificial hybridization between the cultivars (as the pollen donor) and the wild populations of Taiwan has been carried out, named “Hua Lien Bitter Gourd No. 2” and “No. 15”, by the Hualien District Agricultural Research and Extension Station (http://www.hdais.gov.tw/bred). The successful artificial hybridization suggested the possibility of natural hybridization, especially for the crop-to-wild introgression by pollen flow. Different estimates of divergence time implied that the long-term duration of the speciation process might be caused by recurrent asymmetric gene flow, an idea that is supported by inferences of both historical and current rates of migrations. In fact, the crop-to-wild introgression is much more substantial than we had previously thought [64]. A serious genomic impact of introgression is the reduction of the reproductive fitness of wild individuals whose variants are replaced by “domesticated alleles” [65]. In our microsatellite study, two loci, which have extremely high (CMBR114-1) and extremely low divergence (CMBR31), were suggested to be evolving under directional selection and balancing selection, respectively. Although these two outliers are most likely due to the hitchhiking effect, the results still indicated that certain loci were either naturally or artificially selected by the domestication process, resulting in fixation or loss of certain alleles. That is, the domestication-related alleles, good or bad, had been successfully incorporated into wild populations by introgression. Although the genetic assimilation might be relaxed by demographic swamping, the recurrent receiving of pollen from crops would speed progress towards gene replacement [24]. Therefore, the crop-to-wild introgression would cause a serious problem of losing native genes. This study provides an empirical case of such phenomenon of gene replacement by asymmetric introgression.

## Figures and Tables

**Table 1:** Genetic diversity and neutrality tests of the chloroplast *atp*B-*rbc*L spacer in the cultivar accession and the Taiwan accession of *Momordica charantia* (gaps are excluded).

Taxa	*N* a	*H* b	*S* c	*θ* d	*π* e	Tajima’s *D* f
The cultivar accession	20	3	2	0.0005	0.0002	−1.2414
The Taiwan accession	7	2	1	0.0004	0.0003	−1.0062
Hybrid	2	1	0	0	0	−1.0488
Total	29	5	4	0.001	0.0011	0.1977

a Number of lines;

b Number of haplotypes;

c Segregating sites;

d Genetic diversity index estimated from segregating sites;

e Nucleotide diversity estimated by site-by-site pairwise comparison;

f None of the values are significant and the null (neutral) hypothesis cannot be rejected.

**Table 2:** Primer sequences, annealing temperature (*T*_m_), and polymorphic type of eleven microsatellite loci and the expected heterozygosity (*H*_exp_), genetic diversity index (*θ*), number of alleles and effective alleles (*N*_a_ and *N*_e_) of eight neutral evolving and polymorphic loci. The forward and reverse primers are denoted in “F” and “R” in front of the primer sequences, respectively.

Locus	Primer set	*T*_m_	Type *	cultivars				wild population (Taiwan)			
	
				*H*_exp_	*θ*	*N*_a_	*N*_e_	*H*_exp_	*θ*	*N*_a_	*N*_e_
CMBR21	F: 5′-AGATTCTGGTTGTTGGGCAG-3′ R: 5′-CAGCGATGATCAACAGAAACA-3′	59 °C	P	0.528	1.506	3	2.062	0.264	2.075	2	1.324
CMBR22	F: 5′-CCAAAACGACCAAATGTTCC-3′ R: 5′-ATACAGACACGCCTTCCACC-3′	56 °C	P	0.554	1.523	5	2.174	0.615	1.613	3	2.333
CMBR30	F: 5′-CACTGCATACACACACATCCA-3′ R: 5′-AAAAGAAGGAGGAGGAGGG-3′	58 °C	P	0.097	5.186	2	1.105	0.440	1.530	2	1.690
CMBR47	F: 5′-ATCCCAACCCATCACTCTCA-3′ R: 5′-TGGGGACAGGTGAGAATATTAGA-3′	59 °C	P	0.738	2.089	6	3.571	0.791	2.527	5	3.769
CMBR57	F: 5′-GCTCTGAAGAGTGGAATGAGAGA-3′ R: 5′-CCATTTGGGAAGTAGGCATC-3′	59 °C	P	0.708	1.917	6	3.226	0.659	1.726	4	2.579
CMBR66	F: 5′-TCAAGCAAAAACCATAATCAGAA-3′ R: 5′-TCCCTTTTCATCATTTCTCTTCA-3′	52 °C	P	0.272	2.026	3	1.361	0.659	1.726	3	2.579
CMBR82	F: 5′-ACGACTCTTGGAAATCGGTC-3′ R: 5′-TTTAGAAAAGAATCACGAAGAGAGC-3′	54 °C	P	0.344	1.717	3	1.504	0.527	1.506	2	1.960
CMBR152	F: 5′-CCCACATTGGTCTCAACAAG-3′ R: 5′-AAAAAATTTGGCATTAGCTATAAAAA-3′	54 °C	P	0.590	1.567	4	2.353	0.440	1.530	2	1.690
CMBR31	F: 5′-AAACAAACCAAACCAAACCG-3′ R: 5′-AAAAAGAAGCGGGAGTAATGA-3′	58 °C	BS	0.610	1.602	4	2.469	0.703	1.896	3	2.882
CMBR114-1	F: 5′-TGCTTTGCCTTAACCGTCTT-3′ R: 5′-TGAGTGCCCAAGATGTTGTC-3′	52 °C	PS	0.097	5.186	2	1.105	0	0	1	1.000
CMBR114-2	F: 5′-TGCTTTGCCTTAACCGTCTT-3′ R: 5′-TGAGTGCCCAAGATGTTGTC-3′	52 °C	M	-	-	-	-	-	-	-	-
CMBR145	F: 5′-TGTGACAATGTGCAACCAG-3′ R: 5′-AAAAATGGTGTTAAACGACATGG-3′	56 °C	M	-	-	-	-	-	-	-	-

* P: polymorphic; M: Monomorphic; PS: positive selection; BS: balancing selection.

**Table 3:** Analysis of molecular variance (AMOVA) between the cultivar accession and the Taiwan accession. The variance explained by differences among accessions and its significance were calculated using probabilities derived from 1000 permutations.

Source of variation	*d.f.*	Sum of Squares	Variance Components	Percentage of Variance	*R*_ST_	*P*
Between accessions	1	104.955	0.528	0.56	0.0056	0.615
Within accessions	52	4887.786	93.996	99.44		
Total	53	4992.741	94.524	100		

**Table 4:** Log-likelihood nested model tests by the full model (*θ*_C_ ≠ *θ*_T_ ≠ *θ*_A_, *m*_1_ ≠ *m*_2_ ≠ 0, where *m*_1_ = *m*_C→T_ and *m*_2_ = *m*_T→C_). Statistical nonsignificance (not rejecting the null model) is indicated in bold.

Model	T	*θ*_C_	*θ*_T_	*θ*_A_	*m*_C→T_	*m*_T→C_	*df*	2LLR †	*P*
Full model									
mode	0.135	0.3314	0.1767	3.3572	9.95	0.01			
95% CI Low	0.065	0.1736	0.0589	1.708	1.61	0.05			
95% CI High	0.545	1.9409	0.6086	29.5471	19.37	18.85			
Null models									
*m*_1_ = *m*_2_	0.1167	0.5634	0.1469	2.1057	3.2863	3.2863	1	4.3693	0.0366
***m*_2_** **= 0**	**0.0625**	**0.2105**	**0.2230**	**2.6982**	**19.9976**	**0.0001**	**1**	**0.0056**	**0.4702**
*m*_1_ = 0	0.0995	0.4138	0.2023	2.5454	0.0001	9.4322	1	14.8471	5.829 × 10^−5^
*m*_1_ = *m*_2_ = 0	0.1785	0.7870	0.4202	1.8314	0.0001	0.0001	2	718.0318	6.03 × 10^−157^
***θ*_C_** **=** ***θ*_T_**	**0.0625**	**0.2163**	**0.2163**	**2.7202**	**19.9992**	**0.0002**	**1**	**0.3680**	**0.5441**
*θ*_C_ = *θ*_T_ = *θ*_A_	0.2213	0.6101	0.6101	0.6101	7.8461	2.8492	2	16.3993	2.747 × 10^−4^
*θ*_C_ = *θ*_T_, *m*_1_ = *m*_2_	0.0902	0.1906	0.1906	2.1566	19.5826	19.5826	2	9.8940	0.0071
*θ*_C_ = *θ*_T_, *m*_1_ = *m*_2_ = 0	0.1785	0.7026	0.7026	1.8250	0.0001	0.0001	3	746.7677	7.58 × 10^−162^
*θ*_C_ = *θ*_T_ = *θ*_A_, *m*_1_ = *m*_2_	0.2213	0.6107	0.6107	0.6107	5.4662	5.4662	3	51.8731	3.19 × 10^−11^
*θ*_C_ = *θ*_T_ = *θ*_A_, *m*_1_ = *m*_2_ = 0	0.1785	0.8188	0.8188	0.8188	0.0001	0.0001	4	803.2170	7.72 × 10^−173^
*θ*_C_ = *θ*_A_	0.2537	1.0702	0.4156	1.0702	7.3973	0.0478	1	5.9515	0.0147
*θ*_C_ = *θ*_A_, *m*_1_ = *m*_2_	0.2980	1.5204	0.3881	1.5204	1.4217	1.4217	2	25.8596	2.42 × 10^−6^
*θ*_C_ = *θ*_A_, *m*_1_ = *m*_2_ = 0	0.1785	0.9223	0.4204	0.9223	0.0001	0.0001	3	759.3913	1.39 × 10^−164^
*θ*_T_ = *θ*_A_	0.2213	0.6338	0.5800	0.5800	7.8300	2.8436	1	15.4141	8.63 × 10^−5^
*θ*_T_ = *θ*_A_, *m*_1_ = *m*_2_	0.2213	0.6347	0.5806	0.5806	5.4683	5.4683	2	50.8823	8.93 × 10^−12^
*θ*_T_ = *θ*_A_, *m*_1_ = *m*_2_ = 0	0.1785	0.7864	0.8914	0.8914	0.0001	0.0001	3	801.3394	1.11 × 10^−173^

† The log-likelihood ratio result from IMa analysis [33] approximates a *χ*^2^ distribution. For models where migration estimates are set to zero, the expected distribution is a mixture, and the 2LLR approximates a 1/2*χ*^2^ distribution [33].

T: unscaled divergent time; *θ*_C_, *θ*_T_, and *θ*_A_: unscaled population size of cultivar accession, Taiwan accession, and ancestors, respetivly; *m*_C→T_ and *m*_T→C_: migration rate from cultivar accession to Taiwan accession and the opposite direction, respectively; df: degrees of freedom; 2LLR denotes the log-likelihood ratio test expressed by two-times difference of logarithmic likelihoods between alternative and null models; P is the one-tailed probability value for the *χ*^2^ test used for LRT.

**Table 5:** Summary of immigration rate between accessions of *Momordica charantia* estimated by IMa [33], MIGRATE-n [34], and BayesAss [35].

	IMa a		MIGRATE-n a		BayesAss a	
Source	Cultivar accession	Taiwan accession	Cultivar accession	Taiwan accession	Cultivar accession	Taiwan accession
Cultivar accession	-	2.655	-	507.5	0.985	0.071
Taiwan accession	0.195	-	52.5	-	0.015	0.929

a Immigration rates were revealed in *m* of IMa [33], *M* (= *m*/*μ*) of MIGRATE-n [34], and the proportion of migrants in the data sets of two accessions in a migration rate of 0.166 (95% CI: 0.008~0.325) of BayesAss [35], respectively.

**Table 6:** Resources and accessions of the *Momordica charantia* in this study.

Species	Strain	Accessions	Sample size	Resource a, b	Abbreviation
*M. charantia* (white cultivar)	Pai Bitter Gourd	TVI006693	6	NPGRC, TARI, COA	BM1
*M. charantia* (white cultivar)	Pin Tung He Tzu Ku Kua No. 8	TVI007217	6	NPGRC, TARI, COA	BM2
*M. charantia* (white cultivar)	Pai Pi Bitter Gourd	TVI007364	6	NPGRC, TARI, COA	BM3
*M. charantia* (white cultivar)	Chin Lien Bitter Gourd	TVI007723	6	NPGRC, TARI, COA	BM4
*M. charantia* (white cultivar)	Pin Tung He Tzu Bitter Gourd	TVI007895	6	NPGRC, TARI, COA	BM5
*M. charantia* (white cultivar)	Ming Hua	TVI006970	6	NPGRC, TARI, COA	BM8
*M. charantia* (white cultivar)	Lin Nei Tzu Liu Chung	TVI009016	5	NPGRC, TARI, COA	BM12
*M. charantia* (white cultivar)	Chiang Men Ta Ting Bitter Gourd	TVI010175	6	NPGRC, TARI, COA	BM16
*M. charantia* (white cultivar)	Ping Tung Li Kang Bitter Gourd	TVI009615	5	NPGRC, TARI, COA	BM18
*M. charantia* (white cultivar)	Small Bitter Melon	TVI009995	5	NPGRC, TARI, COA	BM20
*M. charantia* (white cultivar)	Chen Chu	TVI006900	6	NPGRC, TARI, COA	BM24
*M. charantia* (white cultivar)	Kuang Han Te Ta Chang Pai Bitter Gourd	TVI009892	5	NPGRC, TARI, COA	BM27
*M. charantia* (white cultivar)	Pai Pi Tsu Mi Bitter Gourd	TVI009534	5	NPGRC, TARI, COA	BM31
*M. charantia* (white cultivar)	Ping Tung Bitter Gourd	TVI007365	5	NPGRC, TARI, COA	BM33
*M. charantia* (white cultivar)	Chinese Gui-Nin No. 2	SYSU-BM-1	5	Exchange from South China Botanical Garden, China.	CBM2
*M. charantia* (green cultivar)	Chin Pi Bitter Gourd	TVI008603	6	NPGRC, TARI, COA	BM22
*M. charantia* (green cultivar)	Chin Pi Bitter Gourd	SYSU-BM-2	6	Exchange from Kunming Institute of Botany, China.	BBM
*M. charantia* (green cultivar)	Kao Mien Bitter Gourd	TVI009317	5	NPGRC, TARI, COA	FBM4
*M. charantia* (wild population from Korea)	Han Ch’eng K’u Kua	CN93MJF135	5	NPGRC, TARI, COA	FBM1
*M. charantia* (wild population from India)	*Momordica charantia* variety	TVI009464	5	NPGRC, TARI, COA	CBM1
*M. charantia* (wild population from Taiwan)	Ping Tung Chiu Chow Bitter Gourd	SYSU-BW-1	8	N 22°39′14″, E 120°38′10″, 205 m altitude	BMW1
*M. charantia* (wild population from Taiwan)	Nan Tou Bitter Gourd	SYSU-BW-2	8	N 23°55′06″, E 120°53′04″, 700 m altitude	BMW2
*M. charantia* (wild population from Taiwan)	Yeh Sheng Bitter Gourd	TVI009560	5	NPGRC, TARI, COA	BMW5
*M. charantia* (wild population from Taiwan)	Hua Lien Yeh Sheng Bitter Melon No. 1	TVI009930	5	NPGRC, TARI, COA	BMW9
*M. charantia* (wild population from Taiwan)	Hua Lien Yeh Sheng Bitter Melon No. 5	TVI009934	5	NPGRC, TARI, COA	BMW10
*M. charantia* (wild population from Taiwan)	Hua Lien Yeh Sheng Bitter Melon No. 10	TVI009959	5	NPGRC, TARI, COA	BMW11
*M. charantia* (wild population from Taiwan)	Ping Tung Bitter Gourd	SYSU-BW-3	8	N 22°24′57″, E 120°39′57″, 686 m altitude	BMW12
Hybrid between wild population and cultivars (*H*)	Hua Lien Bitter Gourd No. 2	HB002	5	HDARES, COA	H2
Hybrid between wild population and cultivars (*H*)	Hua Lien Bitter Gourd No. 15	HB015	5	HDARES, COA	H15

a NPGRC, TARI, COA: The National Plant Genetic Resources Center, Taiwan Agricultural Research Institute, Council of Agriculture of Taiwan, respectively.

b HDARES, COA: Hualien District Agricultural Research and Extension Station, Council of Agriculture of Taiwan.
